# Advanced strategies for production of soy-processing enzyme

**DOI:** 10.3389/fbioe.2022.1042001

**Published:** 2023-01-09

**Authors:** S. M. Mahfuzul Islam, Lu-Kwang Ju

**Affiliations:** Department of Chemical, Biomolecular, and Corrosion Engineering, The University of Akron, Akron, OH, United States

**Keywords:** *Aspergillus niger*, enzyme production, pectinase, α-galactosidase, cellulase, soybean hull

## Abstract

Enzyme production is critical and often costly for biorefinery. It is challenging to produce enzymes with not only high titers but also proper combinations of all required activities in a single fermentation. This work aimed at improving productivity and composition of the multiple enzyme activities required for hydrolysis of complex soybean carbohydrate in a single fermentation. A previously selected *Aspergillus niger* strain was used for its high carbohydrases and low protease production. Strategies of fed-batch substrate addition and programmed pH-decrease rates were evaluated. Cheap soybean hull (SH) was confirmed to induce production of all necessary carbohydrases. Surprisingly, fed-batch SH addition, originally thought to sustain substrate-inducer availability and reduce feedback repression by sugars, did not increase pectinase and cellulase production significantly and even lowered the α-galactosidase production, when compared with batch fermentation having the same total SH amount (all added initially). On the other hand, the pH-decrease rate could be effectively optimized for production of complex enzyme mixtures. The best fermentation was programmed to lower pH from 7 to 4 in 84 h, at a drop rate of .0357 per h. It produced the highest pectinase (19.1 ± .04 U/mL), α-galactosidase (15.7 ± .4 U/mL), and cellulase (.88 ± .06 FPU/mL). Producing these high enzyme activities in a single fermentation significantly improves the effectiveness and economics of enzymatic soy processing, which, e.g., can hydrolyze the 30%–35% carbohydrate in soybean meal to sugars, with minimal protein degradation, to generate high-value protein-rich products and a hydrolysate as fermentation feedstock.

## 1 Introduction

Biorefinery relies on a range of technologies to convert renewable resources to products or building blocks for other products. Enzyme hydrolysis is the most environmentally friendly technology to convert the carbohydrate in biomass to monomeric sugars, which can support production of biofuels and other chemicals by fermentation. However, the process requires optimal mixtures of carbohydrase activities to tackle the biomass structure and carbohydrate composition of the specific biomass source (Mosier et al., 2005; Islam et al., 2018). Soybean, as a major food and industrial crop, is the biomass source considered in this work.

The global soybean production in Year 2020/21 was 363 million metric tons (USDA Foreign Agricultural Service, 2021). Soybean contains 18%–20% oil, about 40% protein, and 25%–30% carbohydrate (Medic et al., 2014). After the oil is extracted, the remaining defatted soybean meal is a solid mixture of mainly protein (∼50%) and carbohydrate (30%–35%) (Medic et al., 2014; Islam et al., 2018). The protein has a good amino acid profile for food and feed uses (Medic et al., 2014). The carbohydrate includes approximately 60% polysaccharides (pectin, hemicellulose, and cellulose) and 40% oligosaccharides (sucrose, stachyose, and raffinose) (Ouhida et al., 2002; Islam et al., 2018). Studies have shown that selective hydrolysis of the carbohydrate in soybean meal while minimizing protein degradation can generate two easily separable product streams: one is the aqueous phase containing mainly hydrolyzed carbohydrate, the other is the wet solid product containing mainly insoluble protein (Loman et al., 2016; Islam et al., 2020). The protein-enriched product has similar protein content to soy protein concentrate or isolate, depending on process conditions, with improved digestibility and much higher value than soybean meal. This process is even more desirable if it also hydrolyzes carbohydrate to monomeric sugars, for use as fermentation substrate for biofuel and biochemical production (Sun and Cheng, 2002; Hasunuma et al., 2013; Loman et al., 2018). Soybean meal pretreatment improves the efficiency of enzymatic carbohydrate hydrolysis (Islam et al., 2018) but the enzyme mixture must also have an adequate composition of multiple activities (pectinase, xylanase, cellulase, α-galactosidase, and sucrase) to hydrolyze the various types of carbohydrate in soybean meal (Loman and Ju, 2016). Economically, it is more advantageous to produce the enzyme mixture in a single fermentation, instead of blending enzymes produced from multiple fermentations.

*Trichoderma reesei* and *Aspergillus niger* are among the most extensively studied fungi for production of these carbohydrases (Castilho et al., 2000; Kolasa et al., 2014; Li et al., 2017). Li et al. (2017) examined 15 *Aspergillus* species and *T. reesei* Rut C30 for enzyme production in shake flasks using cheap soybean hull as inducing substrate (Li et al., 2017). *A. niger* NRRL 322 was one of the two chosen strains that produced minimal protease (140.5 ± 2.1 BAEE U/mL) and maximal carbohydrases (101.7 ± 1.5 U/mL xylanase, 6.36 ± .32 U/mL pectinase, 4.50 ± .03 U/mL α-galactosidase, 3.35 ± .14 U/mL sucrase, and .31 ± .01 FPU/mL cellulase). Using enzyme from such strains for soybean meal/flour processing showed good (∼90%) monomerization of carbohydrate with minimal proteinase-effected degradation (mainly on β-conglycinin α’/α and glycinin acidic 37-kDa subunits) and recovery of most of the proteolytic products by heat-induced precipitation (Islam et al., 2020).

Since high xylanase was already produced in shake flasks, the focus in this work was to improve production of the other carbohydrases by *A. niger* NRRL 322 in pH and dissolved oxygen (DO) controlled fermentors. We evaluated the effects of several nutrient and environmental conditions, including soybean hull concentration, N-sources concentration, fed-batch hull addition, initial pH, and controlled gradient/rate of pH decrease along the fermentation (i.e., having different pH drop rates per h). Fed-batch hull addition was considered for its potential effects of 1) increasing/distributing the substrate and inducer availability to sustain longer active enzyme synthesis and 2) reducing the feedback repression by unconsumed hydrolytic products (sugars), assuming slower sugar generation and accumulation from hydrolysis of the hull added in a smaller quantity each time. The pH effects (initial and decreasing rate) were included in the study because *A. niger* had been reported to have different optimal pH for producing different carbohydrases (Sohail et al., 2009; Ajayi et al., 2018; Li et al., 2018; Elshafei et al., 2022). The objective of this work was to produce enzymes with high pectinase, α-galactosidase, cellulase, and sucrase in a single fermentation.

## 2 Materials and methods

### 2.1 Materials

*A. niger* NRRL 322 was obtained from the United States Department of Agriculture (USDA) Agricultural Research Service (ARS) Culture Collection. The culture was maintained on potato dextrose agar (30 g/L, Sigma, P2182). Soybean hull was provided by the Archer Daniels Midland Company (Decatur, IL). Soybean hull was reported to have 9%–14% protein (on moisture free basis) (Mielenz et al., 2009), i.e., about 1.4%–2.2% N (converted using the general protein-to-N ratio of 6.25). The total reducing sugar content of soybean hull was measured as 64 ± 2% by weight and the monomeric sugar contents were: glucose, 35.7 ± 1.3%; xylose, 13.2 ± .8%; galactose, 5.9 ± .5%; arabinose, 6.5 ± .2%; and mannose, 5.7 ± 1.4% (Islam et al., 2017). The main equipment used in this study included a UV-visible spectrophotometer (Shimadzu UV-1601, Colombia, MD); a shaker (Thermo Scientific MaxQ 5000 Incubating/Refrigerating floor shaker, Ashville, NC); two fermentors with controls for pH, DO, agitation, and temperature (BioFlo 110, NewBruswick Scientific, Edison, NJ); a water bath (Boekel Scientific ORS-200); and a micro centrifuge (Eppendorf Centrifuge 5415D). Proteose peptone was purchased from Remel Microbial Products (division of Thermo Fisher Scientific) with 10% total (Kjeldahl) nitrogen. Other chemicals, unless otherwise specified, were purchased from Sigma Aldrich (St. Louis, MO).

### 2.2 Preculture and general fermentation conditions

Inoculum was prepared by adding three loops of cells from a mature agar plate to 150 mL preculture medium in a 500 mL shake flask and incubating for 48 h in a shaker at 25°C and 200 rpm. (At 48 h, pH reached or neared the lowest level of ∼3.5, allowing no or minimal further cell growth.) The preculture medium, modified from the Mandels and Weber medium (Coffman et al., 2014), contained 20 g/L soybean hull, 2.0 g/L KH_2_PO_4_, 1.4 g/L (NH_4_)2SO_4_, 1.0 g/L proteose peptone, .3 g/L urea, .4 g/L CaCl_2_·2H_2_O, .3 g/L MgSO_4_·7H_2_O, .2 g/L Tween 80, 5 mg/L FeSO_4_·7H_2_O, 2 mg/L CoCl_2_·2H_2_O, 1.6 mg/L MnSO_4_·H_2_O, and 1.4 mg/L ZnSO_4_·7H_2_O. The fermentation, made in stirred tank fermentors with 1–1.5 L working volume, was inoculated with 10% (v/v) preculture and controlled for DO, pH, agitation, temperature, and foaming. The fermentation medium differed from the above preculture medium only in the soybean hull and non-hull N-sources (NH_4_)2SO_4_, proteose peptone, and urea) concentrations and types of addition (i.e., batch or fed-batch) as described in the following subsections. The soybean hull and non-hull N-sources concentrations used in the preculture medium are hereafter referred to as 1x, to allow easier description of the higher fold (2x, 3x, etc.) concentrations used in some other fermentations. Note that 1x non-hull N sources provided .54 g/L total N while 1x soybean hull had .29–.45 g/L total N. Therefore, soybean hull was not only the C source but also a potential N source if most of hull N could be consumed by the fungal cells. Conditions for different fermentations are summarized in Table 1. DO was maintained above 20% (air saturation) by automatic oxygen addition to the .5 VVM aeration with filter-sterilized air. At the later stage of fermentation, nutrient depletion would cause DO to increase even without oxygen supplementation; all fermentations were terminated/harvested when DO reached 70%–80% at 120–192 h, depending on the fermentation conditions studied. pH was either uncontrolled or controlled by specific designs, as described in the following subsections. Agitation speed was 350–450 rpm and temperature was controlled at 25°C, according to a previous study (Li et al., 2020). Foaming was controlled by automatic antifoam addition (Trans-278, Trans-Chemco, Inc., Bristol, WI). Daily samples were taken for enzyme analysis. Samples were centrifuged at 10,000 *g* for 10 min to remove the solids (cells and remaining soybean hull) and the supernatants collected were stored at -20°C prior to analysis. While it was strongly desirable to follow the fungal cell concentration during the fermentation, solid soybean hull used as the main substrate made it impossible to measure cell dry-weight concentration. Attempts to measure intracellular protein and DNA concentrations (Callow and Ju, 2012) were also unsuccessful because of the protein and DNA introduced with hull.

**TABLE 1:** Fermentations made with different pH and soybean hull and non-hull N-sources concentrations and addition schemes.

Fermentation	pH		N-sources	Soybean hull concentration		
	Initial	Control	Concentration	Initial	Addition	Total
F1	6	No	2x	2x	No	2x
F2	7	No	2x	2x	No	2x
F3	6	No	2x	5x	No	5x
F4	7	No	5x	5x	No	5x
F5	6	No	2x	2x	1x @72 h	3x
F6	6	No	2x	2x	1x @48, 72, 96 h	5x
F7	7	Drop to 5.5 @96 h^a^	2x	5x	No	5x
		Rate: .0156/h				
F8	7	Drop to 4.2 @96 h^a^	2x (+1x)^b^	5x	No	5x
		Rate: .0292/h				
F9	7	Drop to 4.0 @84 h^a^	2x	5x	No	5x
		Rate: .0357/h				

Notes: All fermentations were made at 25°C, 350–450 rpm, and DO ≥ 20% (by O_2_ supplementation).

^a^ After the period (84 or 96 h) of controlled pH decrease, pH was not controlled.

^b^ Initial non-hull N-sources concentration was 2x; .25x addition at 24, 36, 48 and 60 h.

### 2.3 Study for effects of initial pH and concentrations of soybean hull and N-sources

Four batch fermentations (F1–F4) were made to serve as basis for comparison with the later fermentations with fed-batch or pH control designs. F1 and F2 had 2x (40 g/L) soybean hull and 2x non-hull N-sources. To explore the potential effect of initial pH, pH was adjusted, after autoclaving, to 6 for F1 and 7 for F2, using 1N HCl or NaOH. pH was then allowed to change without control: decreasing initially due to, e.g., production of organic acids and consumption of ammonia, and increasing later due to, e.g., ammonia release from endogenous metabolism. Both were harvested after 120 h. F3 had the same 2x N-sources and initial pH 6 as F1 but a much higher 5x (100 g/L) soybean hull concentration. Comparison of results from F1 and F3 was intended to show the effect of higher soybean hull concentration on enzyme production in batch fermentations without pH control. To be compared with F2 (and F1 and F3, if initial pH effect was insignificant), F4 was done with initial pH 7 but much higher concentrations in both (5x) soybean hull and (5x) N-sources, to investigate the effect of both higher C and N concentrations. Because the higher soybean hull concentration supported longer cell metabolism, the harvest times were longer in F3 (171 h) and F4 (137 h), longer for F3 presumably because of the lower cell concentration supported by the lower N-sources concentration.

### 2.4 Study for effects of fed-batch soybean hull addition

Two fed-batch fermentations F5 and F6 were made with the same initial pH (6) and N-sources concentration but different schemes of soybean hull addition. Both had 2x soybean hull initially, sterilized by autoclaving. Later, F5 was added with another batch of 1x soybean hull at 72 h while F6 was added with 3 batches of soybean hull, 1x each time at 48, 72, and 96 h, respectively. (These addition times were chosen based on the results from the above batch fermentations, which showed enzyme production started around 24 h and maxed around 120 h (described in the Results and Discussion section); so, 72 h was the midpoint while 48, 72, and 96 h divided the enzyme production period into 4 equal intervals.) The soybean hull added later was sterilized by dry heat (160°C for 3 h), to avoid increasing liquid volume. The total soybean hull concentration was 3x in F5 and 5x in F6. Correspondingly, the final fermentation broth was harvested at 120 h for F5 and 190 h for F6, when the DO increased above 70%. Results of the fed-batch fermentations F5 and F6 were designed to be compared with the results of batch fermentations F1 and F3, which had soybean hull concentrations at the low (1x) and high (5x) ends investigated, to show the effects of fed-batch soybean hull addition.

### 2.5 Study for effects of controlled pH-drop rate

The effects of 3 different pH-drop rates were evaluated. The batch fermentations F7–F9 had the same initial pH (7), soybean hull concentration (5x), and N-sources concentration (2x); this condition was found best in the batch fermentations. Previous batch fermentations typically lasted for 120–144 h, with enzyme production slowed or stopped after 72–96 h. Accordingly, these 3 fermentations with controlled pH-drop rates were also designed to run for 144 h, with pH control for up to 96 h (leaving the remaining 48+ h without pH control). The pH drop rate was programmed and controlled by automatic addition of 1N HCl/NaOH. F7 was made with the slowest pH drop rate of .0156 per h, decreasing pH from 7 (initial) to 5.5 at 96 h. In previous studies, pH 5.5–6.5 was found to be more favorable for pectinase production by *Aspergillus* (Li et al., 2018; Li et al., 2020). F7 was therefore designed to maximize pectinase production by 96 h. However, the high pH was not optimal for synthesis of α-galactosidase, xylanase, and especially cellulase (Sohail et al., 2009; Ajayi et al., 2018; Li et al., 2018; 2020; Elshafei et al., 2022). Higher pH drop rates were therefore used in F8 and F9, to lower the pH to about 4 in 96 h (F8) or 84 h (F9). pH 4 was optimal for cellulase production by *Aspergillus* (Sohail et al., 2009; Li et al., 2018) while pH lower than 4 was unfavorable for production of any of the carbohydrases considered in this study. Accordingly, the controlled pH-drop rate in F8 was increased to .0292 per h, which lowered the pH to 4.2 at 96 h, and the rate in F9 was further increased to .0357 per h but only until 84 h, when pH already dropped to 4.0 (to avoid the unfavorably lower pH). F8 also had additional N-sources supplementation: .25x each, at 24, 36, 48 and 60 h, to explore whether the supplementation could increase enzyme production by periodical stimulation of cell growth/activities and/or provision of N sources for enzyme synthesis.

### 2.6 Enzyme analysis

Extracellular activities of cellulase, pectinase, α-galactosidase, and sucrase were measured. Analysis was made with triplicate samples; the obtained average and standard deviation was reported. One unit of enzyme activity corresponds to the activity that gives the target product at a rate of 1 μmol/min. The target product concentration was determined by the 3,5-dinitrosalicylic acid (DNS) test method using different reducing sugars as standards, except for the α-galactosidase analysis.

Cellulase FPU was analyzed using a modified method of Ghose (Ghose, 1987; Coffman et al., 2014) after adjusting the sample cellulase activity to .05–3 FPU/mL. The analysis procedure was as follows: (1) Cut Whatman No. 1 filter paper into pieces of 6 × 1 cm (∼50 mg/piece). Roll and insert a piece (1 cm in height) into a 25 mL test tube. Add 1.4 mL .05 M sodium citrate buffer (pH 4.8) and 100 µL sample to the tube, to completely immerse the filter paper. (2) Prepare the blank in the same way but without the filter paper. (3) Incubate the sample and blank in a water bath at 50°C for 1 h. (4) Add 3 mL regular DNS solution (10 g/L DNS, 16 g/L NaOH, and 300 g/L sodium potassium tartrate) to each tube to stop the enzyme reaction. (5) Incubate the DNS-added tubes in boiling water for 10 min. (6) Add deionized water to make the total volume 25 mL, mix, and then measure the absorbance of reaction supernatant at 540 nm with a spectrophotometer. Cellulase activity was calculated using the following equation by determining the amount (mg) of reducing sugar released using a calibration equation established with pure glucose solutions as standards.

$$Cellulase\;\left(FPU/mL\right)=\frac{glucose\;released\;\left(mg\right)}{\left(60\;\min\right)\left(0.1\;mL\;enzyme\;sample\right)}\times \frac{1\;mmol}{180\;mg}\times \frac{1000\;\mu mol}{1\;mmol}=0.926\times glucose\;released\;\left(mg\right).$$

The pectinase method was modified from that of Li et al. (2015). The sample was diluted to .3–.7 U/mL pectinase and measured using the following procedure: (1) Prepare the substrate solution/suspension by mixing .5 g citrus pectin in 100 mL .1 M sodium citrate buffer (pH 4.8). Heat the mixture under stirring until vapor appears but no boiling. Turn off heating, stir the mixture overnight, and then store the substrate mixture at -20°C for future use. (2) Add 100 µL sample and 900 µL substrate mixture to a 25 mL test tube. (3) Prepare the (enzyme-free) blank with only 900 µL substrate. (4) Incubate the sample and blank in a water bath at 50°C for 30 min. (5) Add 3 mL DNS solution (without sodium potassium tartrate, to avoid precipitation) to each tube and add 100 µL sample to the blank (to account for the sample-associated turbidity). DNS analysis was then done to determine the amount (mg) of reducing sugar released, using D-galacturonic acid (monohydrate) solutions as standards. The pectinase activity was calculated using the following equation:

$$Pectinase\;\left(\frac{U}{mL}\right)=1.57\times galacturonic\;acid\;released\;\left(mg\right)$$

Sucrase was assayed using a modified method of Uma et al. (2010). The method was best for samples adjusted to .2–2.0 U/mL sucrase. The procedure was very similar to that for pectinase, with the following differences: (1) sucrose was used for preparing the substrate solution (without heating and extended mixing); (2) the enzyme reaction at 50°C was allowed for 20 min; and (3) the regular (tartrate-containing) DNS solution was used. Glucose standards were used for the DNS analysis calibration. The sucrase activity was calculated as:

$$Sucrase\;\left(\frac{U}{mL}\right)=2.78\times glucose\;released\;\left(mg\right).$$

The α-galactosidase assay used a modified method of Kumar et al. (2012). Samples were diluted to .05–.2 U/mL α-galactosidase. The procedure was as follows: (1) Prepare the substrate solution by dissolving .033 g p-nitrophenyl-α-D-galactopyranoside in 100 mL .1 M sodium citrate buffer (pH 4.8). (2) Mix 100 µL sample with 900 µL substrate solution. (3) Prepare the blank with 900 µL substrate solution. (4) Incubate sample and blank at 50°C for 10 min. (5) Add 2 mL .5 M sodium carbonate (pH 9.8) to sample and blank to stop the reaction and develop the color from released p-nitrophenol. (6) Add 100 µL sample to the blank. (7) Measure the absorbance at 405 nm. Calibration was done with pure p-nitrophenol standards, to determine the enzyme-released p-nitrophenol. The α-galactosidase activity was calculated by the following equation:

$$\alpha -Galactosidase\;\left(\frac{U}{mL}\right)=7.19\times p-nitrophenol\;released\;\left(mg\right)$$

## 3 Results and discussion

### 3.1 Enzyme production in batch fermentations without pH control

**FIGURE 1:**
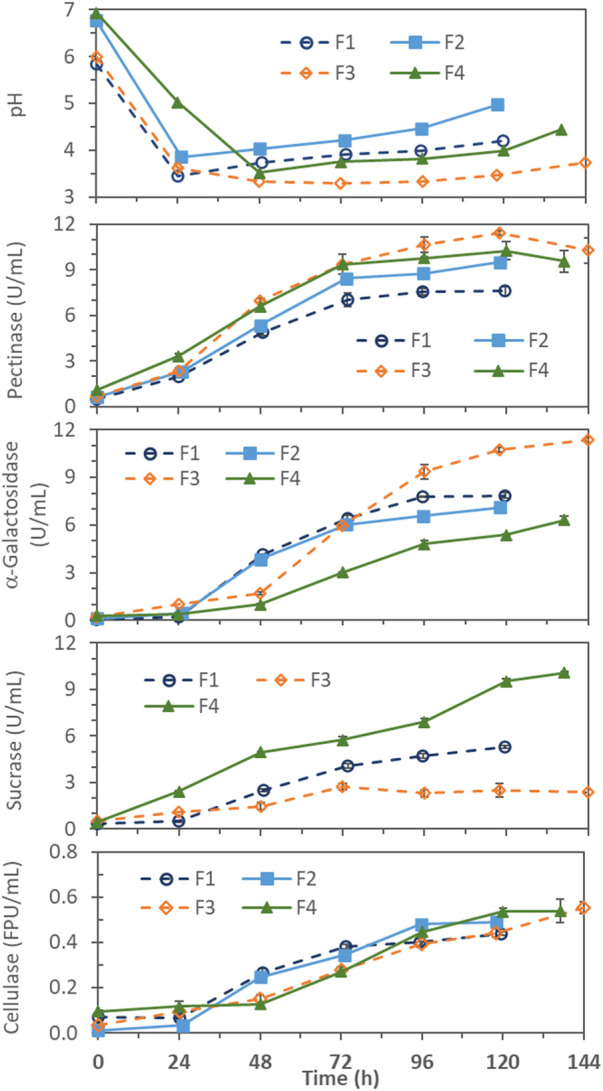
pH and enzyme production profiles in pH-uncontrolled batch fermentations with different soybean hull (SH) and non-hull N-sources (N) concentrations and initial pH; F1 = 2x SH, 2x N, pH 6; F2 = 2x SH, 2x N, pH 7; F3 = 5x SH, 2x N, pH 6; and F4 = 5x SH, 5x N, pH 7. 1x SH = 20 g/L soybean hull and 1x N = 1.4 g/L (NH_4_)2SO_4_, 1.0 g/L proteose peptone, and .3 g/L urea.

Profiles of enzyme production and pH change are compared in Figure 1 for the 4 batch fermentations (F1–F4) made without pH control. pH decreased to the lowest value of 3.3–3.8 by 24–48 h. As expected, the lowest pH values were slightly lower in the F1 and F3 (pH_min_ 3.3–3.4) with lower initial pH (6), than those in the F2 and F4 (pH_min_ 3.5–3.8) with higher initial pH (7). For F1 and F2, made with 2x (40 g/L) soybean hull and 2x non-hull N sources (containing 1.08 g N), pH started to rise after about 24 h, suggesting the rate of monosaccharide generation by enzymatic hydrolysis was no longer sufficient to support active growth (pH decreasing) metabolism of cells, and the endogenous (pH increasing) metabolism became more dominant after that stage of fermentation. For F3 and F4, the higher (5x) initial soybean hull feed provided a larger amount of substrate to hydrolyze for monosaccharide generation and delayed the pH rise to 48 h in F4 and 72–96 h in F3. F3 was made with only 2x non-hull N sources (same as F1 and F2) while F4 with 5x N sources (having same hull-to-N sources ratio as F1 and F2). The much larger hull-to-N sources ratio of F3 preserved more hull to sustain non-growth activities (including carbohydrase enzyme production) and delayed the onset of pH rise to a much later time (72–96 h).

Among the enzymes measured, pectinase production generally occurred first. This might be because pectin forms the outer layer of the carbohydrate matrix that makes up the cell wall in soybean. Production of α-galactosidase began about 1 day after the pectinase production, hypothetically induced by the intermediate oligosaccharides released from pectin hydrolysis (Li et al., 2018). Cellulase production started after pH had dropped substantially. It was reported that endoglucanase and β-glucosidase (two major components of cellulase) were optimally produced at pH 4.0 in solid-state fermentation of *A. niger* MS82 on grass- and corn-based lignocellulosic materials (Sohail et al., 2009). Cellulase production in submerged fermentation of *Aspergillus foetidus* on soybean hull was also reported to be optimal at pH 4 (after better cell growth was established at higher pH) (Li et al., 2018).

#### 3.1.1 Effect of initial pH

Regarding the effect of initial pH, results from F1 and F2, which differed only in the initial pH (6 vs. 7), were found to be quite comparable for α-galactosidase and cellulase (Figure 1). Only pectinase production differed appreciably, reaching 9.51 ± .19 U/mL in F2 and 7.60 ± .30 U/mL in F1. The finding is consistent with the report that pectinase production by *Aspergillus* on soybean hull favored higher pH (6-7) (Li et al., 2018).

#### 3.1.2 Effects of soybean hull concentration and soybean hull-to-N ratio

F1 and F3, with the same initial pH 6, differed only in the initial soy hull concentration (2x vs. 5x) and, accordingly, the hull-to-N ratio (2x:2x vs. 5x:2x). As shown in Figure 1, the higher soy hull concentration at also higher hull-to-N ratio supported more cellulase, pectinase and α-galactosidase production in F3: 0.55 ± 0.03 FPU/mL cellulase, 11.4 ± 0.4 U/mL pectinase, and 11.4 ± 0.1 U/mL α-galactosidase, than the 0.44 ± 0.01 FPU/mL cellulase, 7.6 ± 0.3 U/mL pectinase, and 7.9 ± 0.2 U/mL α-galactosidase in F1. F2 and F4, both with initial pH 7, also differed in the initial soy hull concentration (2x vs. 5x) but F4 also had a higher 5x N-sources concentration (vs. 2x in F1, F2 and F3). Therefore, F2 and F4 had the same hull-to-N ratio. In this case, the higher soybean hull concentration at the same hull-to-N ratio in F4, compared to F2, gave only slightly higher maximum pectinase (10.3 ± .6 vs. 9.5 ± .2 U/mL), comparable cellulase (.54 ± .05 vs. .49 ± .02 FPU/mL), and even slightly lower α-galactosidase (6.3 ± .3 vs. 7.1 ± .1 U/mL). The larger hull-to-N ratio (5x:2x) was found to be significantly more favorable for pectinase, α-galactosidase, and cellulase production in batch fermentations.

Interestingly, the hull-to-N ratio had an almost opposite effect on sucrase production. F1 and F4, with the same ratio of soybean hull to N-sources concentrations (2x:2x and 5x:5x), showed continuously increasing sucrase production, reaching 5.3 ± .1 U/mL in F1 and 10.1 ± .05 U/mL in F4. On the other hand, in the F3 with a much higher hull-to-N ratio (5x:2x), sucrase production stopped at 96 h, reaching only 2.7 ± .2 U/mL. These observations could be explained by two potential mechanisms. First, sucrase production might be growth-dependent, and N-sources became growth-limiting in F3 after 96 h causing the sucrase production to stop. Growth-dependency of sucrase production has been reported for fungi, e.g., *Aspergillus nidulans* (Vainstein and Peberdy, 1991) and *Thermomyces lanuginosus* (Maheshwari et al., 1983), where sucrase production depended on the synthesis of total protein, DNA, and RNA. Second, sucrase production was feedback-repressed by sugar accumulation, due to faster generation (by the higher carbohydrase activities already produced and more (5x) soybean hull substrate provided in F3) than consumption (by the slower non-growing cell metabolism in F3). This feedback repression of sucrase synthesis by various sugars, particularly glucose, has been well documented, e.g., for *Saccharomyces cerevisiae*, *Neurospora crassa*, and *A. nidulans* (Vainstein and Peberdy, 1991). In separate fermentations made in this laboratory (unpublished results), this *A. niger* strain was grown on soy molasses-based media; cells, while growing well, produced only very low levels of sucrase in presence of inducers (sucrose and raffinose (Maheshwari et al., 1983; Vainstein and Peberdy, 1991) and likely stachyose). The finding supported that sucrase synthesis by this *A. niger* strain was feedback-repressed by sugars.

High soybean hull concentration also had effects on fermentation operation. At lower agitation speeds, a significant portion of the 5x soybean hull tended to settle and become poorly available to cells and enzymes. With increased agitation to suspend the soybean hull, the hull particles tended to cause more foaming and more attachment of cells and hull on the fermentor wall above liquid. More attention for defoaming was needed to reduce the associated delays in pH decrease and enzyme production.

### 3.2 Enzyme production in fed-batch fermentations without pH control

**FIGURE 2:**
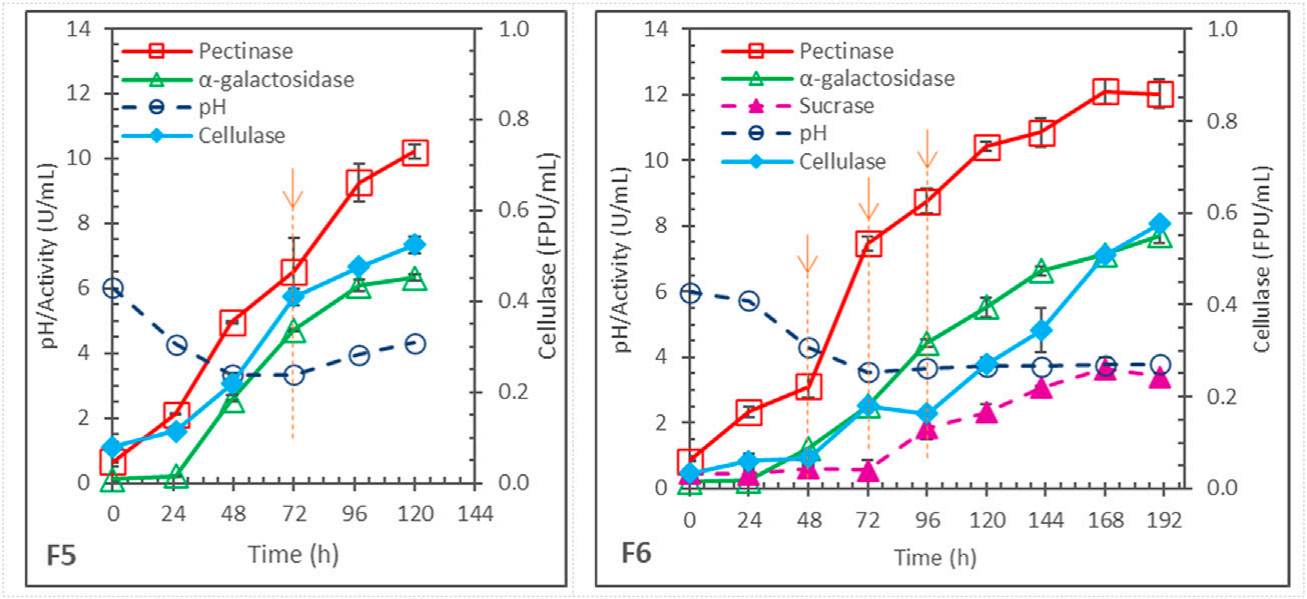
Enzyme production in pH-uncontrolled, fed-batch fermentations with same initial pH 6 and 2x N-sources but different soybean hull (SH) addition; each arrow with dashed line indicating 1x SH addition; F5 = 2x SH initially + 1x SH at 72 h, and F6 = 2x SH initially + 1x SH each at 48, 72, and 96 h.

In the above batch fermentations, after 72 h, pectinase production almost stopped in F1, F2 and F4, and significantly slowed in F3. The cause was likely the depletion of inducer. Therefore, the effects of fed-batch addition of soybean hull were studied in two fermentations (F5 and F6) with the same initial pH 6 as in F3 (and F1). Results are shown in Figure 2.

F5 differed from F1 only in the addition of 1x more soybean hull at 72 h. While pectinase production stopped after 72 h in F1 (Figure 1), it continued in F5 and reached 10.2 ± .2 U/mL at 120 h (significantly higher than the 7.6 ± .3 U/mL pectinase obtained in F1). Cellulase production also increased from .44 ± .01 FPU/mL in F1 to .52 ± .02 FPU/mL in F5. However, F5 produced less α-galactosidase than F1, 6.32 ± .11 vs. 7.85 ± .15 U/mL.

In F6, 1x soybean hull was added 3 times, at 48, 72, and 96 h. Continual production of pectinase occurred after each addition and extended the production till 168 h, reaching 12.1 ± .4 U/mL. However, this was not significantly higher than the maximum of 11.4 ± .4 U/mL pectinase observed in F3 (*p* = .10, >.05), which was a batch fermentation with the same total 5x soybean hull and 2x non-hull N-sources (differing only in the batch vs. fed-batch hull addition.) Cellulase production was also similar between F3 (.55 ± .03 FPU/mL) and F6 (.58 ± .01 U/mL). On the other hand, the α-galactosidase production was significantly worse in the fed-batch F6 (7.7 ± .2 U/mL at 190 h) than in the batch F3 (11.4 ± .1 U/mL at 168 h).

Fed-batch hull addition was originally hypothesized to distribute the substrate and inducer availability better over the fermentation time and to reduce the feedback repression by sugars, because of the possibly lower generation rates from hydrolysis of hull added in a smaller quantity each time. However, the hypothesis was not supported by the experimental results. Instead, the results suggested that the dynamic generation and consumption of inducers and repressors might be somewhat self-regulated by the fungal cell metabolism, and the fed-batch hull addition could disturb the condition and negatively affect the enzyme synthesis. In any case, the results highlighted the difficulty in managing the transient induction and repression, particularly for α-galactosidase synthesis, in this process using complex soybean hull as substrate for producing mixtures of enzymes.

### 3.3 Enzyme production in batch fermentations with controlled pH decreases

**FIGURE 3:**
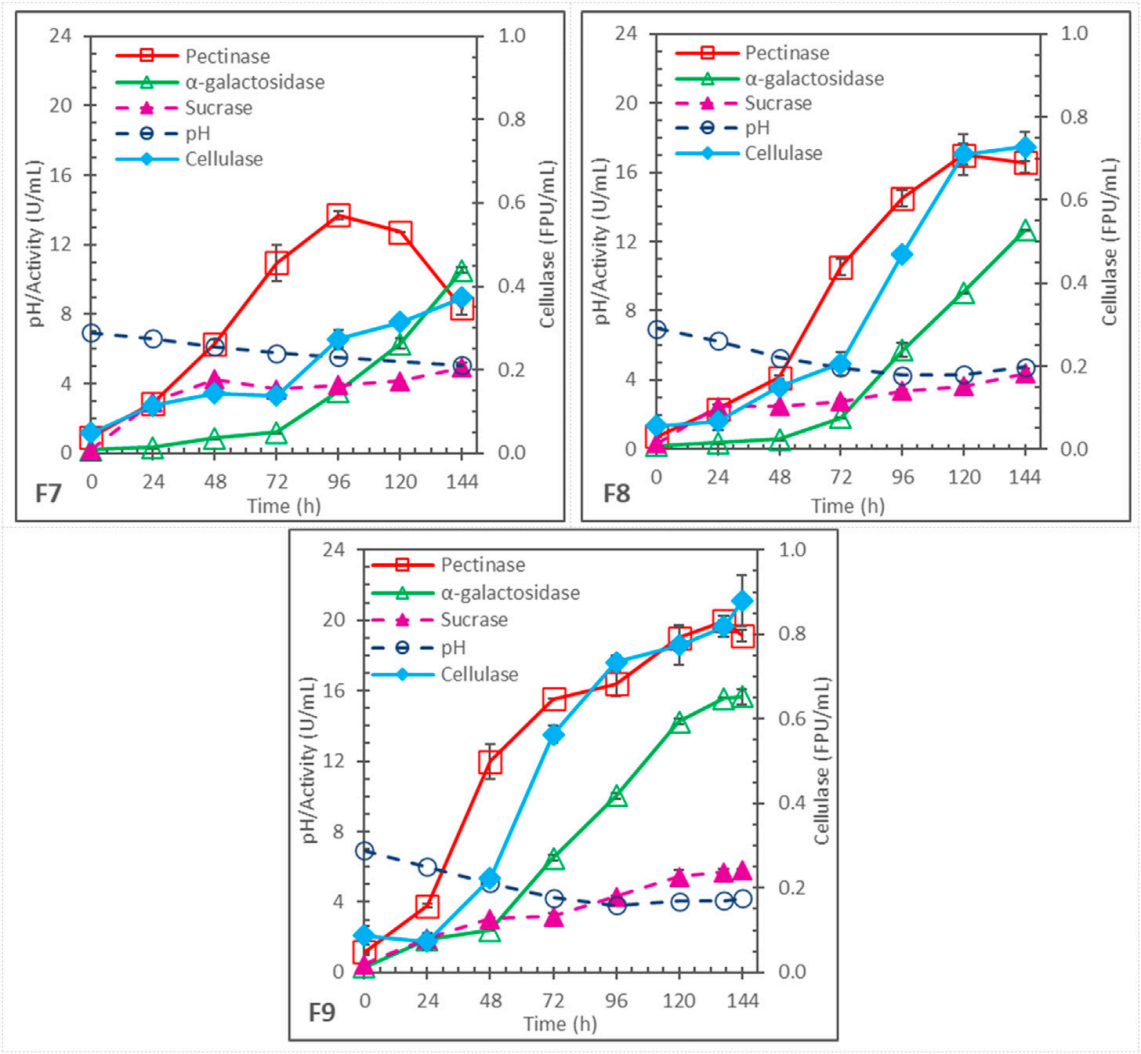
Enzyme production in batch fermentations with same initial pH 7, 5x soybean hull, and 2x initial N-sources but with different controlled pH-drop rates and durations; F7: pH-drop rate = .0156/h, to pH 5.5 at 96 h; F8: pH-drop rate = .0292/h, to pH 4.2 at 96 h; and F9: pH-drop rate = .0357/h, to pH 4.0 at 84 h.

In the fermentations F1–F6 described above, pH was allowed to change naturally, and pH dropped rapidly to 3.3–4 in the first 24–48 h. The low pH could be undesirable for subsequent enzyme production and different carbohydrases have different optimal pH for production by *Aspergillus* (Sohail et al., 2009; Ajayi et al., 2018; Li et al., 2018; 2020; Elshafei et al., 2022). Three different schemes of controlled pH decrease were examined in F7, F8, and F9. The schemes are summarized in Table 1 and the pH profiles and enzyme production profiles are shown in Figure 3. These fermentations had the same initial pH 7 but different controlled pH-drop rates: .0156 pH drop/h (to pH 5.5 at 96 h) for F7, .0292 pH drop/h (to pH 4.2 at 96 h) for F8, and .0357 pH drop/h (to pH 4.0 at 84 h) for F9. After the end (96 or 84 h) of controlled pH decrease, pH was left to change naturally. F7 and F9 had 5x soybean hull and 2x N-sources, the same as F3. F8 also had 5x soybean hull and 2x N-sources initially but was added with 4 batches of .25x N-sources at 24, 36, 48, and 60 h, respectively. The N-sources additions were intended to investigate whether they could support additional cell growth and significantly affect the enzyme production; however, the results were inconclusive. On the other hand, the different pH-drop rates had significant effects: higher enzyme production, compared to the pH-uncontrolled F3, was found in F8 and F9 for all the measured carbohydrases. F9, with the highest pH drop rate among the 3 rates evaluated, performed particularly well, and produced 19.1 ± .04 U/mL pectinase, 15.7 ± .4 U/mL α-galactosidase, .88 ± .06 FPU/mL cellulase, and 5.8 ± .1 U/mL sucrase.

In F3, pH dropped to a very low pH of 3.6 by 24 h while the pH in F7, F8 and F9 were all controlled to drop much slower. Apparently, the low pH (below 4) was less favorable for carbohydrase production; by slowing down the pH decrease, more enzymes were produced at their higher optimal pH for longer periods. However, when the pH was controlled to drop too slowly, the substrate hydrolysis and consumption by cells might be too fast, causing early exhaustion of substrate and inducers. This is suggested by the early decline of pectinase after 96 h in F7 and after 120 h in F8. Similar pectinase degradation in the later substrate-limiting stage of *Aspergillus* fermentation on soybean hull was observed in previous studies, particularly when pH was relatively high (Li et al., 2018; Li et al., 2020). Among the systems investigated, F9 provided the best compromise that allowed longer periods of active enzyme production without too fast carbohydrate hydrolysis.

### 3.4 Discussion and comparison of enzyme production at different fermentation conditions

Soybean hull could support growth of *A. niger* and induce synthesis of various carbohydrase enzymes useful for enzymatic soybean processing. However, soybean hull is a complex substrate. Its hydrolysis during fermentation generates not only the intermediates that induce enzyme synthesis but also the monosaccharide end products that can accumulate and repress further enzyme synthesis if not consumed fast enough by cells for growth and other metabolic activities. The dynamic induction-repression and generation-consumption relationships are expected to change with changing cell growth/metabolic rate and cell, substrate, and enzyme concentrations. In addition, different carbohydrases have different optimal pH for their synthesis. Managing all these factors for improving enzyme production in batch fermentation is challenging. In this study, soybean hull-to-N ratio was varied to observe how the extent of cell growth (which decreases with increasing hull-to-N ratio) would affect enzyme production and, accordingly, to identify the more suitable hull-to-N ratio. Fed-batch soybean hull addition was attempted in two fermentations to observe the potential of this strategy in managing the transient induction-repression and generation-consumption relationships. Finally, controlled pH decrease was investigated as a strategy to provide extended periods of different optimal pH for production of different enzymes, instead of letting pH drop quickly to too low levels without control.

**FIGURE 4:**
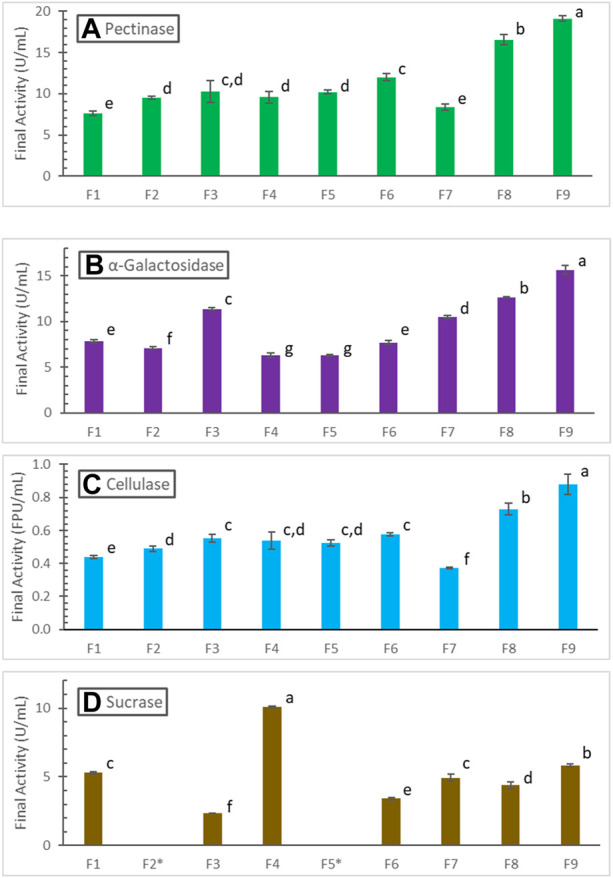
Comparison of **(A)** pectinase, **(B)** α-galactosidase, **(C)** cellulase, and **(D)** sucrase production in different fermentations (see conditions in Table 1; sucrase not measured in F2 and F5). Activities are grouped and labeled according to the Tukey’s pairwise comparisons, with insignificantly different activities sharing the same letter label.

Figure 4A summarizes the final pectinase activities obtained in all the fermentations studied in this work. The lowest 7.6 ± .3 U/mL pectinase was found in the batch fermentation F1 where 2x soybean hull and 2x N-sources were used with initial pH 6. The final pectinase activity, 8.3 ± .4 U/mL, in F7 (with 5x soybean hull, 2x N-sources, initial pH 7, and slowest controlled pH-drop rate of .0156/h till 96 h) was insignificantly different from that of F1, based on Tukey’s pairwise comparison method (*α* = .05). However, this was due to significant degradation of pectinase in F7 after reaching substrate limitation; pectinase in F7 peaked at a much higher 13.7 ± .2 U/mL at 96 h as shown in Figure 3. Other fermentations gave 25%–152% higher pectinase production compared to F1. Fed-batch soybean hull addition improved pectinase production by 34.3% (F5, with 3x soybean hull in total) and 58.1% (F6, 5x soybean hull), respectively. However, the batch fermentation F3 with 5x soybean hull also gave 50% higher pectinase, insignificantly different from F6 according to the Tukey’s test (Figure 4A). Therefore, while the positive effect of providing more hull in the fermentation was significant, the effect of fed-batch addition *per se* was not statistically significant. Properly controlled pH decreases were much more effective in improving pectinase production; the highest improvements were found in F8 (117.9%, with a pH-drop rate of .0292/h) and F9 (151.5%, with a pH-drop rate of .0357/h), both being batch fermentations with 5x soybean hull. The highest pectinase activity obtained was 19.1 ± .4 U/mL in F9.

The trend of final cellulase produced in different fermentations, summarized in Figures 4C is similar to that of pectinase. The highest cellulase activity of .88 ± .06 FPU/mL was also found in F9, representing a 100% increase over that in F1. The trend for α-galactosidase, summarized in Figures 4B is not as similar; the fed-batch soybean hull addition in F5 and F6 gave even lower α-galactosidase production. Overall, fed-batch hull addition was not an effective strategy to manage the dynamic generation and consumption of inducers and repressors, and it might even negatively affect the enzyme synthesis. Non-etheless, properly controlled pH decreases were again effective in improving α-galactosidase production. The highest α-galactosidase found in F9 was 15.7 ± .4 U/mL, corresponding to a 99.9% increase over that in F1.

The trend of final sucrase activities (Figure 4D) is very different from those of pectinase, cellulase and α-galactosidase. The highest sucrase activity of 10.1 ± .1 U/mL was found in the batch fermentation F4 with 5x soybean hull and 5x N-sources. The batch fermentation F3 with 5x soybean hull and 2x N-sources gave the lowest 2.4 ± .1 U/mL sucrase, about 55% lower than that produced in F1 with 2x hull and 2x N-sources. Fed-batch hull addition (F6) and pH-decreasing-rate control (F7, F9) used in other fermentations with 5x hull and 2x N-sources improved sucrase production over F3; non-etheless, only the best case F9 reached a sucrase activity comparable to F1. The ratio of hull to N-sources (lowest in F1, 2x:2x, and F4, 5x:5x), which dictates when or if cell growth would be limited by N availability, was found to be an important factor to sucrase production.

Overall, the novelty of this work was in effectively producing an enzyme with multiple activities at proper composition in a single fermentation. While all were required for processing a complex biomaterial such as soybean meal, these activities were expressed under different optimal conditions and were subject to induction and repression, possibly with hydrolytic intermediates/products of one activity serving as the inducers and/or repressors of the other activities. The dynamic, unclear complexity makes it extremely challenging to develop an effective process for producing the enzyme with multiple activities at proper composition in a single fermentation. Through a series of well-planned experiments, the main contribution achieved by this work was development of an effective enzyme production process using the special pH control scheme, which enabled significant increase of enzyme productivity and adjustment of enzyme composition.

There are hundreds of reports on using *A. niger* to produce carbohydrases. The predominant majority of those using submerged fermentation focused on producing one carbohydrase or the component enzymes in one carbohydrase group, e.g., β-glucosidase, endo- and/or exo-glucanase of cellulase. Some produced one of the carbohydrases examined in this study plus another enzyme, e.g., cellulase and xylanase (Kim et al., 1997; Villena and Gutierez-Correa, 2012) and sucrase and inulinase (Dinarvand et al., 2017). Three reports were found to produce 2 of the 4 examined carbohydrases by submerged fermentation of *A. niger*, and none produced 3 or 4 of the carbohydrases (Kumar et al., 2011; Wang et al., 2019; Li et al., 2020). The *A. niger* strains, C sources, and fermentation conditions used as well as the maximal activities reported in these 3 reports are summarized in Table 2 and compared with those of this work. The rarity of previous work on producing multiple enzymes in a single submerged *A. niger* fermentation, for processing of complex materials, lends support to the novelty of this work. The much higher enzyme activities obtained in this work substantiate the significance of the advanced fermentation strategy developed in this study.

**TABLE 2:** Comparison of multi-carbohydrase activities^a^ produced by submerged fermentation of *Aspergillus niger*.

Strain	C Source	Condition	Pectinase	α-Galactosidase	Cellulase	Sucrase	Ref
NRRL 322	40 g/L soy hull	Stir-tank fermentor; no pH control, pH_0_ = 6	7.6	7.8	—	—	Li et al. (2020)
NCIM 548	65 g/L wheat bran, corn bran and kinnow peel (2:1:2)	Shake flask, pH_0_ = 4.8	1.6	—	.36	—	Kumar et al. (2011)
Gyx 086	30 g/L wheat straw	Shake flask (50 mL), pH_0_ = 5	6.3^b^	—	.43**^c^**	—	Wang et al. (2019)
NRRL 322	100 g/L soy hull	Stir-tank fermentor; pH_0_ = 7, with controlled pH drop rate	19.1	15.7	.88	5.8	This work

Note:

^**a**^ Activities reported in FPU/mL for cellulase and U/mL for all others.

^**b**^ Reported for polygalacturonase, instead of the composite pectinase.

^**c**^ Reported for carboxymethyl cellulase, instead of cellulase.

## 4 Conclusion

The study confirmed that *A. niger* NRRL 322 fermentation could be used to produce the carbohydrases (pectinase, α-galactosidase, sucrase, and cellulase) required for enzymatic soy processing. Using a higher (5x:2x) ratio of soybean hull (SH) to N-sources concentrations in the fermentation medium, instead of 2x:2x or 5x:5x, was found desirable, for producing more α-galactosidase (a limiting carbohydrase) instead of sucrase. Fed-batch SH addition was effective in increasing pectinase and cellulase production, but it decreased α-galactosidase production. The most effective approach was to properly control the pH-decrease rate. Using this approach with a pH-decrease rate of .0357 per h (dropping from pH 7 to 4 in 84 h) and with a medium containing 5x SH and 2x N-sources, the fermentation produced the highest pectinase (19.1 ± .04 U/mL), α-galactosidase (15.7 ± .4 U/mL), and cellulase (.88 ± .06 FPU/mL) activities. The study results can significantly improve the effectiveness and economics of enzymatic soybean processing.

## Data Availability

The raw data supporting the conclusion of this article will be made available by the authors, without undue reservation.
